# Ionic liquids-ultrasound based efficient extraction of flavonoid glycosides and triterpenoid saponins from licorice†

**DOI:** 10.1039/c8ra01056k

**Published:** 2018-04-16

**Authors:** Shuai Ji, Yujie Wang, Zhenyu Su, Dandan He, Yan Du, Mengzhe Guo, Dongzhi Yang, Daoquan Tang

**Affiliations:** Department of Pharmaceutical Analysis, School of Pharmacy, Xuzhou Medical University 209 Tongshan Road Xuzhou 221004 China tdq993@hotmail.com +86 516 83263133 +86 516 83263133; Jiangsu Key Laboratory of New Drug Research and Clinical Pharmacy, School of Pharmacy, Xuzhou Medical University 209 Tongshan Road Xuzhou 221004 China tangdq@xzhmu.edu.cn

## Abstract

Flavonoid glycosides and triterpenoid saponins are the main chemical constituents of licorice. In this study, an ionic liquids-ultrasound based extraction (IL-UAE) method was established to simultaneously extract liquiritin (LQ), liquiritin apioside (LA), isoliquiritin (ILQ), isoliquiritin apioside (ILA) and glycyrrhizic acid (GA) from licorice. A series of 1-alkyl-3-methylimidazolium ILs with different anions and alkyl chain lengths of cations were investigated and compared, and 1-butyl-3-methylimidazolium acetate ([C_4_MIM]Ac) was finally selected as the extractant. The extraction parameters of the IL-UAE procedure were optimized, and the established method was validated in linearity, stability, precision, repeatability and recovery. The IL-UAE approach exhibited much higher extraction efficiency comparing with conventional UAE, and needed shorter extraction time and smaller solvent to solid ratio comparing with the pharmacopoeia method. In addition, the microstructures of licorice powders were observed before and after extraction with help of a scanning electron microscope (SEM) in order to explore the extraction mechanism. The results suggested that ILs as green solvents were effective for extraction of flavonoid glycosides and triterpenoid saponins from licorice.

## Abstract 2

An ionic liquids-ultrasound based method for efficient extraction of flavonoid glycosides and triterpenoid saponins from licorice was established.

## Graphical abstract

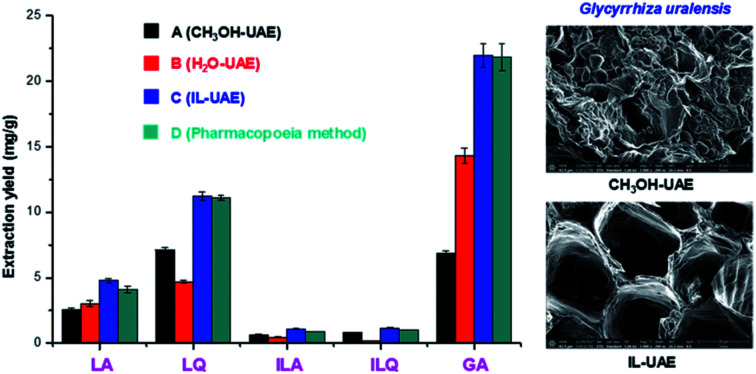

## 1. Introduction

Licorice, the dried roots and rhizomes of *Glycyrrhiza uralensis* Fisch. (Leguminosae) and related species, is one of the most popular herbal medicines with a medicinal history of at least 2000 years worldwide.^1^ It is mainly used to treat coughs, influenza and liver damage in clinical practice, and has been recorded in the pharmacopoeia of China and other countries.^2^ The major chemical constituents of licorice include flavonoid glycosides, triterpenoid saponins and free flavonoids, and the former two types possess the much higher content in licorice according to our recent research.^3^ Thereinto, liquiritin (LQ), liquiritin apioside (LA), isoliquiritin (ILQ) and isoliquiritin apioside (ILA) are the main flavonoid glycosides with the total content of approximately 32 mg g^−1^ in the dried herb materials of licorice, and glycyrrhizic acid (GA) is the main triterpenoid saponin with the content of approximately 15 mg g^−1^.^3^ The five compounds possess various biological activities including neuroprotective, antioxidant, antifungal, antigenotoxic, antineoplastic, anti-inflammatory and antiviral activities.^4–8^ Thus, they are often used as index components of licorice,^9^ and efficient extraction of the five compounds will contribute to quality control of licorice.

Traditionally, flavonoid glycosides and triterpenoid saponins in licorice are extracted mainly by decocting, refluxing and ultrasonic wave.^10–12^ Nevertheless, these methods have many drawbacks such as time-consuming, low extraction efficiency, using of toxic and hazardous organic solvents and bad environment conditions. Recently, environment friendly techniques catch more attraction with the development of the *Green Chemistry*.^13^ Ionic liquids (ILs), composed of organic cations and inorganic or organic anions, have been proposed as green alternatives to organic solvents. They are liquid near room temperature, and have negligible volatility, low flammability, chemical stability, wide liquidus range, and good solubility and extracting ability for organic compounds.^14–16^ Moreover, a large number of possible variations in cation and anion allow the fine-tuning of the ILs properties.^17^ Therefore, ILs have been applied as solvent in the preparation of various useful substances from plant samples such as alkaloids, saponins and phenolic compounds through direct extraction, aqueous biphasic systems, liquid–liquid extraction, liquid-phase microextraction and solid-phase microextraction.^18–21^ Yang *et al.* used an ionic liquids-based ultrasonic-assisted extraction method to extract three compounds from licorice, and obtained high extraction efficiency. The three compounds represented all three types of chemical constituents in licorice namely a free flavonoid, a flavonoid glycoside and a triterpenoid saponin.^22^ So far, there is no literature of specific extraction of main flavonoid glycosides and triterpenoid saponins from licorice using ILs, and the relevant extraction mechanism has never been explored.

In this study, an ionic liquids-ultrasound based extraction (IL-UAE) method was established to simultaneously extract the main flavonoid glycosides (LA, LQ, ILA and ILQ) and triterpenoid saponins (GA) from licorice (the roots of *Glycyrrhiza uralensis*). The influential parameters of the IL-UAE procedure were optimized systematically, and the extraction efficiency was evaluated by determination of the five target compounds in the extracts by HPLC-UV analysis. Moreover, several conventional UAE and the pharmacopoeia method were compared with the established IL-UAE approach. In order to explore the extraction mechanism, the microstructures of licorice powders before and after extraction were investigated by scanning electron microscopy (SEM).

## 2. Experimental

### 2.1 Chemicals and materials

**Fig. 1:**
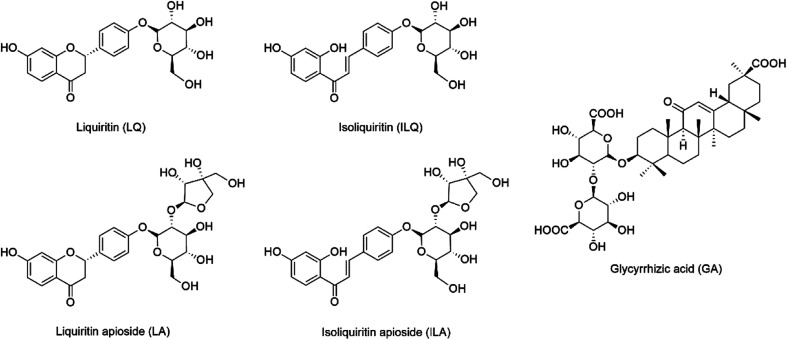
Chemical structures of liquiritin (LQ), liquiritin apioside (LA), isoliquiritin (ILQ), isoliquiritin apioside (ILA) and glycyrrhizic acid (GA).

LQ, LA, ILQ, ILA and GA were purchased from Sichuan Weikeqi Biological Technology Co., Ltd. (Chengdu, China), and their structures are shown in Fig. 1. Their purities were above 95% according to HPLC-UV analysis. All ionic liquids ([C_2_MIM]Br, [C_4_MIM]Ac, [C_4_MIM]Cl, [C_4_MIM]BF_4_, [C_4_MIM]NO_3_, [C_4_MIM]HSO_4_, [C_4_MIM]Br, [C_6_MIM]Br, [C_8_MIM]Br and [C_10_MIM]Br, where C_2_MIM = 1-ethyl-3-methylimidazolium, C_4_MIM = 1-butyl-3-methylimidazolium, C_6_MIM = 1-hexyl-3-methylimidazolium, C_8_MIM = 1-octyl-3-methylimidazolium, C_10_MIM = 1-decyl-3-methylimidazolium) were gained from Chengjie Chemical Reagents Co., Ltd. (Shanghai, China), and their structures are shown in Table S1.† HPLC grade methanol, acetonitrile and formic acid were obtained from Mallinkrodt Baker (Phillipsburg, NJ, USA), and de-ionized water was acquired by an arium pro-water purification system (Sartorius, Göttingen, Germany). All the other reagents were obtained commercially.

The scanning electron microscope (SEM) pictures, used to evaluate the morphology of the licorice powder after extraction, were acquired using a field emission gun Hitachi S-4800 scanning electron microscope with a 5 kV acceleration voltage (Hitachi Co. Ltd., Tokyo, Japan).

### 2.2 Plant material

The roots and rhizomes of licorice (*Glycyrrhiza uralensis*) were collected by the authors in September 2012 in Chifeng City, Inner Mongolia Autonomous Region, People's Republic of China. It was identified by one of us (D. Tang). A voucher specimen (no. GC-201220) has been deposited at School of Pharmacy, Xuzhou Medical University, Xuzhou, People's Republic of China.

### 2.3 Ionic liquids-ultrasound based extraction

For IL-UAE, SB-5200D ultrasonic bath (Ningbo Scientz Biotechnology Co. Ltd., Ningbo, China) with the power of 300 W was used, and 40 KHz transducers were annealed at the bottom of the rectangular container. The temperature was controlled at room temperature by the replacement between inlet and outlet water. The raw materials of licorice were pulverized into fine powder, and then sieved with stainless steel sieves to 30 mesh for use. ILs were dissolved in water at different concentrations, and 1.0 g of accurately weighed licorice powder was mixed with 10 mL of different kinds of IL solutions in a 50 mL flask. The suspensions were then extracted in an ultrasonic water. The optimum anions, cations, concentrations of ILs, soaking time, extraction time and solvent to solid ratio were systematically investigated by a series of single factor tests. After extraction, the extract solutions were centrifuged and filtered through a 0.22 μm nylon membrane before HPLC analysis. All samples were prepared and analyzed in triplicate. The extraction yields of LQ, LA, ILQ, ILA and GA were defined as follows:1
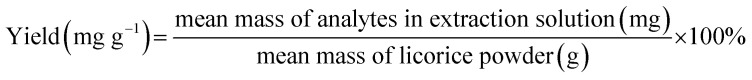
where mean mass of licorice powder referred to the average mass of three samples before extraction. The mean mass of analytes in extraction solution was analyzed by HPLC.

### 2.4 Conventional reference extraction

Methanol and water were selected as the reference solvent in the UAE of flavonoid glycosides and triterpenoid saponins in licorice, and the extraction experiments were operated under the optimized conditions for each of the extraction solvent. Briefly, 1.0 g of licorice powder were mixed with 10 mL of methanol and water in a 50 mL flask, and the suspensions were extracted in an ultrasonic water. Both extract solutions were filtered through 0.22 μm membrane filter before direct injection into the HPLC system.

### 2.5 Preparation of standard solutions

Standard stock solutions of LA, LQ, ILA, ILQ and GA were prepared by directly dissolving them in 1.5 M [C_4_MIM]Ac. The concentrations of the five compounds in stock solutions were 1.07 mg mL^−1^ (LA), 1.43 mg mL^−1^ (LQ), 0.21 mg mL^−1^ (ILA), 0.21 mg mL^−1^ (ILQ) and 3.21 mg mL^−1^ (GA), respectively. Working standard solutions were obtained by appropriate dilution with 1.5 M [C_4_MIM]Ac, and all the standard solutions were stored at 4 °C before analysis.

### 2.6 HPLC analysis

HPLC analysis was performed on an Agilent 1260 HPLC system including a quaternary pump, a diode array detector, an autosampler and a column compartment (Agilent, USA). Samples were separated on an Eclipse XDB-C_18_ column (4.6 × 250 mm, 5 μm) protected with a Zorbax Extend-C_18_ guard column (4.6 × 12.5 mm, 5 μm). The column temperature was 35 °C. The mobile phase consisted of acetonitrile (A) and water containing 0.1% (v/v) formic acid (B). A linear gradient elution program was used as follows: 0 min, 15% A; 25 min, 55% A; 30 min, 95% A; 35 min, 95% A. A 10 min post-run time was set to fully equilibrate the system. The flow rate was 1.0 mL min^−1^. UV spectra were obtained by scanning from 200 nm to 400 nm, and the samples were detected at 254 nm (LQ, LA and GA) and 365 nm (ILQ and ILA). The sample injection volume was 2 μL.

## 3. Results and discussion

### 3.1 Optimization of ionic liquids-ultrasound based extraction (IL-UAE)

#### 3.1.1 Selection of ionic liquids

Recently, imidazolium-based ILs consisting of various *N*,*N*′-dialkylimidazolium cations have become one of the most promising ILs. Among them, 1-alkyl-3-methylimidazolium-based ILs are usually used in the extraction of various kinds of natural products.^23^ As known, the structures of ILs have significant influences on their extraction efficiency for analytes, owing greatly to their distinct multiple interactions with analytes and dissolving ability for target compounds.^24^ Thus, a series of 1-alkyl-3-methylimidazolium-based ILs aqueous solutions with different cations and anions were tested in this study to select the optimal ILs as the extraction medium of LQ, LA, ILQ, ILA and GA from licorice.

##### 3.1.1.1 Selection of cations

**Fig. 2:**
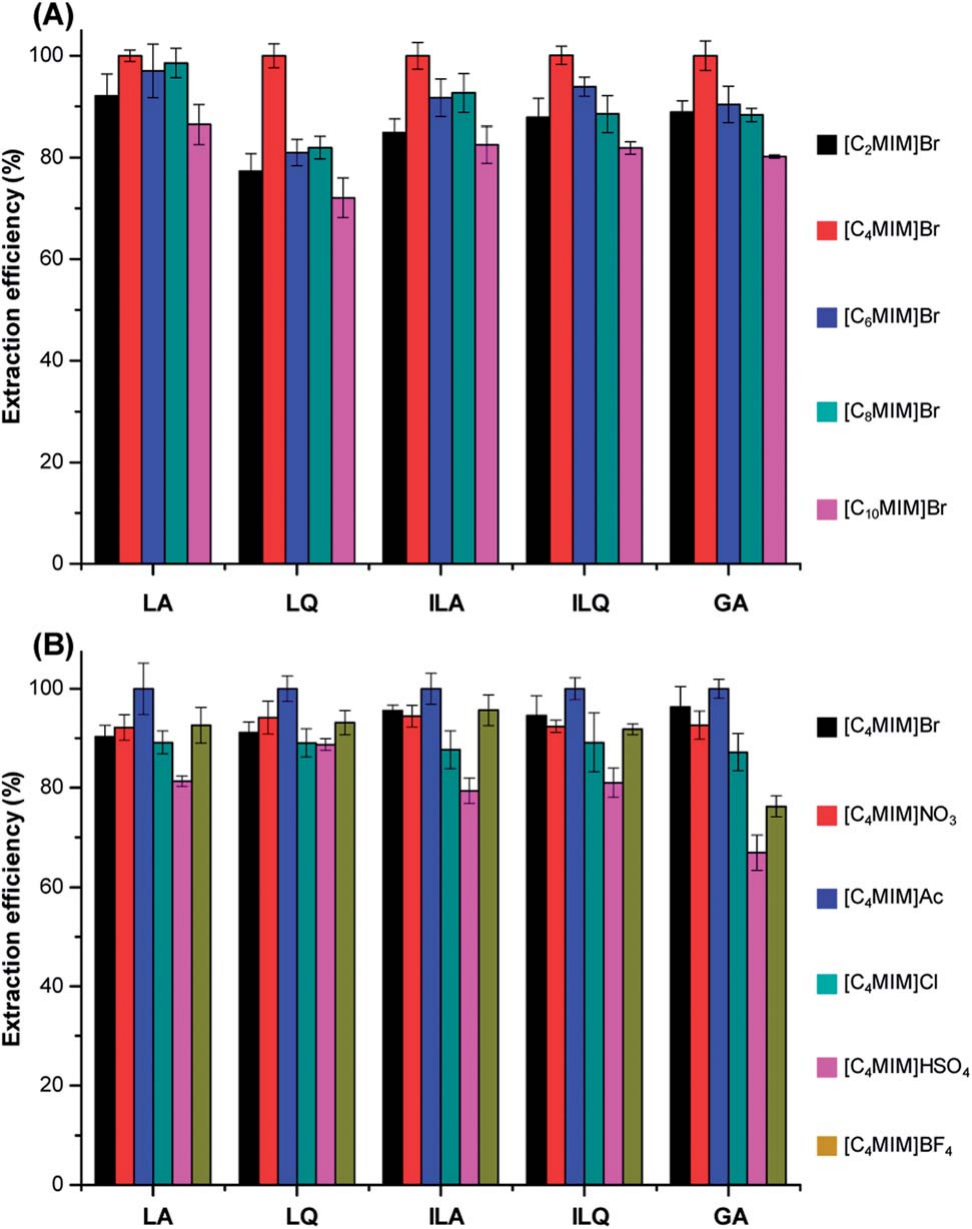
Effect of ILs with different anions (B) and alkyl chain lengths of cations (A) on the extraction efficiency of five target analytes from licorice. The extraction efficiency is expressed as the percent of observed values to max observed value of each target analyte.

A series of 1-alkyl-3-methylimidazolium cations, including [C_2_MIM]^+^, [C_4_MIM]^+^, [C_6_MIM]^+^, [C_8_MIM]^+^ and [C_10_MIM]^+^, were used to investigate the effects of the alkyl chain length on the UAE efficiency for target analytes from licorice. We chose Br^−^ as the same anion since it usually showed good performance based on previous literatures.^22,23^ The extraction process was performed under the same conditions: licorice powder of 1.0 g, solvent to solid ratio of 10 : 1 mL g^−1^, ILs concentrations of 1.5 M and extraction time of 20 min. The results are readily observed from Fig. 2A that the extraction efficiency of flavonoid glycosides and triterpenoid saponins increased when the alkyl chain length increased from ethyl to butyl. However, the extraction efficiency gradually decreased with further increase of the alkyl chain length.

[C_4_MIM]Br showed higher extraction efficiency than [C_2_MIM]Br probably due to the stronger solvation for target compounds, since the hydrogen bond acidity for cations increased from ethyl to butyl.^24^ The hydrophobicity and viscosity enhanced with increasing of the alkyl chain length of cations according to literatures.^23^ Therefore, the extraction efficiency of hydrophilic LA, LQ, ILA, ILQ and GA decreased when alkyl chain further increased, based on the similarity-intermiscibility theory. According to these results, [C_4_MIM]^+^ was used as the optimal cation.

##### 3.1.1.2 Selection of anions

In order to gain higher extraction efficiency of LA, LQ, ILA, ILQ and GA, the [C_4_MIM]^+^-based ILs with six kinds of different anions [Br^−^, NO_3_^−^, CH_3_COO^−^(Ac^−^), Cl^−^, HSO_4_^−^ and BF_4_^−^] were investigated in UAE under the same extraction conditions as Section 3.1.1.1. All these ILs were hydrosoluble. The results illustrated in Fig. 2B showed that the anions could significantly influence the extraction yields. Acetate was more efficient than the other ILs for all the target compounds, probably due to its stronger multi-interactions including π–π, ionic/charge–charge and hydrogen bonding with those compounds, resulting in higher dissolution of them. On the other hand, HSO_4_^−^ exhibited the lowest extraction efficiency. The above results suggested that the extraction yields of flavonoid glycosides and triterpenoid saponins were both cation- and anion-dependent for the same type of ILs.

#### 3.1.2 Optimization of [C_4_MIM]Ac concentration

**Fig. 3:**
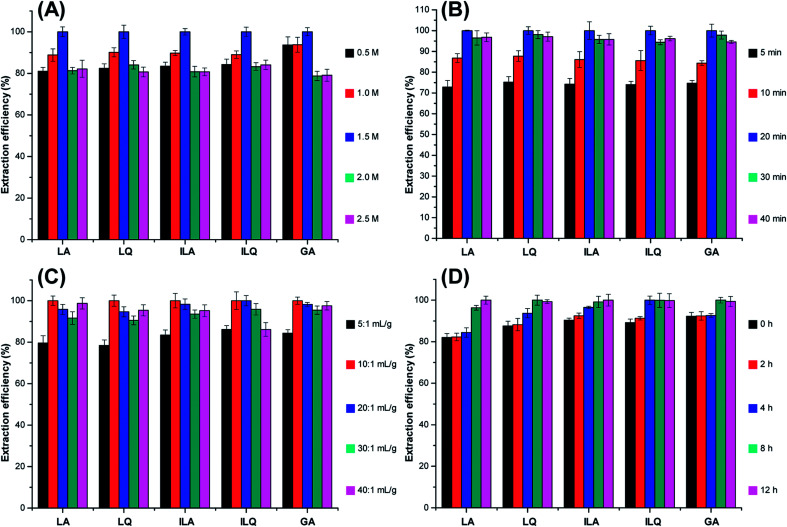
Effect of [C_4_MIM]Ac concentration (A), ultrasonic time (B), solvent to solid ratio (C) and soaking time (D) on the extraction efficiency of five target analytes from licorice. The extraction efficiency is expressed as the percent of observed values to max observed value of each target analyte.

Concentrations of ILs have effect on extraction efficiency of target analytes. In order to find out the optimal IL concentration for UAE of LA, LQ, ILA, ILQ and GA, extraction was performed in IL aqueous solution of different concentrations (from 0.5 M to 2.5 M). In the light of Fig. 3A, the extraction efficiency of flavonoid glycosides and triterpenoid saponins increased significantly when the concentration of [C_4_MIM]Ac was increased from 0.5 M to 1.5 M. However, the extraction efficiency decreased with further increased [C_4_MIM]Ac concentration from 1.5 M to 2.5 M. This might be because the high viscosity of the solvent at high IL concentrations led to poor infiltration of the solvent into the plant tissues, which resulted in decreased extraction efficiency of target compounds. Thus, 1.5 M of [C_4_MIM]Ac was selected for the subsequent experiments.

#### 3.1.3 Optimization of extraction time

Generally, higher extraction efficiency requires longer extraction time. In order to optimize extraction time, licorice powder (1.0 g) was extracted with different ultrasonic time (5, 10, 20, 30 and 40 min) using 10 mL of [C_4_MIM]Ac aqueous solution. The results obtained by the HPLC analysis in Fig. 3B indicated that the average extraction efficiency of LA, LQ, ILA, ILQ and GA increased with extended ultrasonic time from 5 min to 20 min. However, when ultrasonic time further extended, the average extraction efficiency slightly decreased. This was probably because some flavonoid glycosides and triterpenoid saponins decomposed, which was also observed in previous study.^25^ Therefore, 20 min was selected as the optimal ultrasonic time for all subsequent experiments.

#### 3.1.4 Optimization of solvent to solid ratio

Superfluously high solvent to solid ratio could cause procedure complex and unnecessary waste, while lower one may result in insufficient extraction efficiency of target compounds. To evaluate the influence of solvent to solid ratio on extraction efficiency of the five target analytes, a series of experiments were performed with different solvent to solid ratios (5 : 1, 10 : 1, 20 : 1, 30 : 1 and 50 : 1 mL g^−1^). As shown in Fig. 3C, the extraction efficiency of LA, LQ, ILA, ILQ and GA increased when the solvent to solid ratio changed from 5 : 1 mL g^−1^ to 10 : 1 mL g^−1^. However, the extraction efficiency slightly decreased when the ratio was above 10 : 1 mL g^−1^. The reasons might be that a lower solvent to solid ratio was enough for extraction, and ultrasonic energy was absorbed and dispersed by a larger volume of solvent, which was disadvantageous to ultrasonic assistant extraction.^26^ Thus, a solvent to solid ratio of 10 : 1 mL g^−1^ was selected as the optimum ratio for sample preparation.

#### 3.1.5 Optimization of soaking time

For herbal medicines, soaking time also has some influence on extraction efficiency of target analytes. To obtain optimal soaking time, licorice powder was soaked in [C_4_MIM]Ac aqueous solution for 0, 2, 4, 8 and 12 h before IL-UAE. As shown in Fig. 3D, extraction efficiency of LA, LQ, ILA, ILQ and GA gradually increased when soaking time increased from 1 h to 8 h, probably due to increased diffusion of the solvent into the cellular structure, which allowed improved solubilization of flavonoid glycosides and triterpenoid saponins. However, the extraction efficiency had no obvious change when the soaking time was further prolonged. Based on this, we chose 8 h as the optimum soaking time.

According to the above results, the optimum IL-UAE conditions for efficient extraction of LA, LQ, ILA, ILQ and GA from licorice were summarized as follows: extraction solvent 1.5 M [C_4_MIM]Ac aqueous solution, extraction time 20 min, solvent to solid ratio 10 : 1 mL g^−1^ and soaking time 8 h.

### 3.2 Comparison of the IL-UAE approach with the reference solvent extraction

The established IL-UAE approach was compared with the conventional UAE process using reference solvents including water (H_2_O-UAE), methanol (CH_3_OH-UAE) and 70% ethanol (70% C_2_H_5_OH-UAE, the pharmacopoeia method) in order to investigate the effect of the use of ILs. According to the preliminary experiments, we selected the best condition for each of the following extraction methods: H_2_O-UAE (extraction time 40 min and solvent to solid ratio 10 : 1 mL g^−1^), CH_3_OH-UAE (extraction time 30 min and solvent to solid ratio 10 : 1 mL g^−1^), and the pharmacopoeia method (extraction time 30 min and solvent to solid ratio 500 : 1 mL g^−1^).

**Fig. 4:**
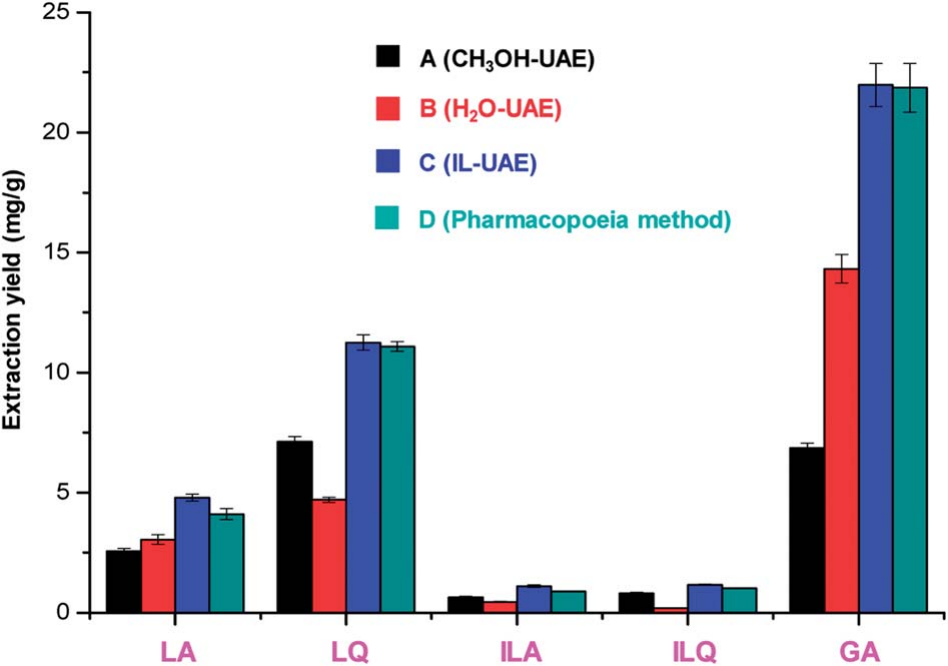
Comparison of IL-UAE with other extraction methods.

**Fig. 5:**
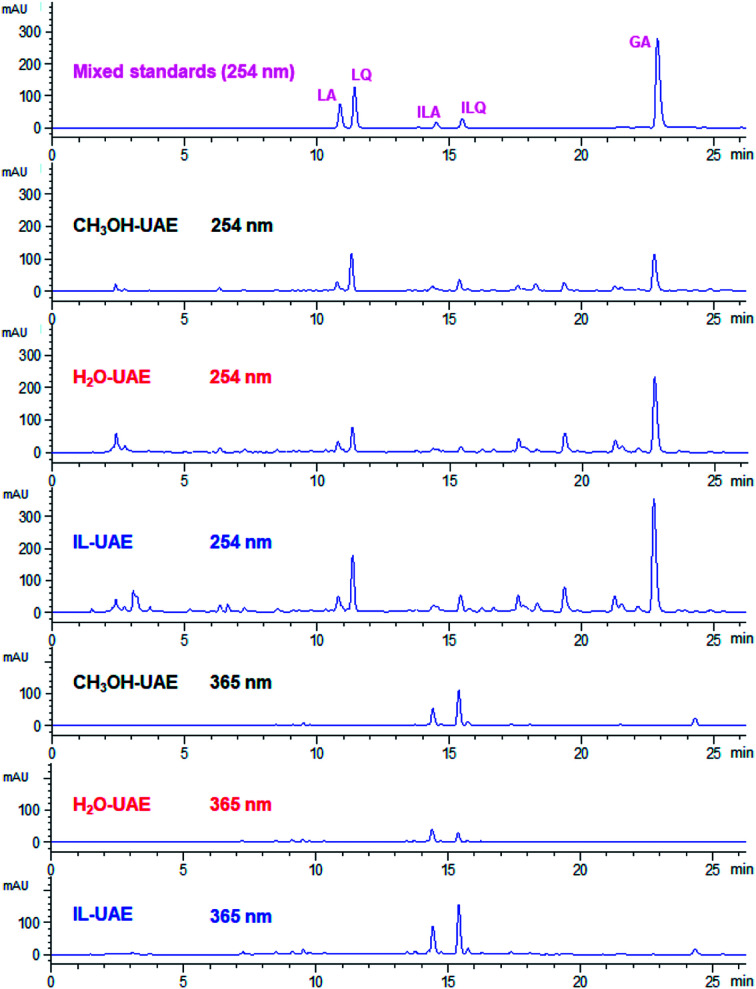
Representative HPLC chromatograms of mixed standards and licorice extract obtained through different extraction methods.

As shown in Fig. 4, the extraction yields of LA, LQ, ILA, ILQ and GA obtained by IL-UAE were 4.79 ± 0.15, 11.25 ± 0.30, 1.11 ± 0.05, 1.17 ± 0.02 and 21.94 ± 0.90 mg g^−1^, respectively, which were much higher than H_2_O-UAE (increase 1.5 to 6.0 times) and CH_3_OH-UAE (increase 1.4 to 3.2 times). Moreover, IL-UAE needed shorter extraction time (20 min *vs.* 30 min) and much smaller solvent to solid ratio (10 : 1 mL g^−1^*vs.* 500 : 1 mL g^−1^) than the pharmacopoeia method. Several representative HPLC chromatograms of licorice extract obtained through different extraction methods are shown in Fig. 5. When toxicity and flammability of methanol are considered, it is clear that IL-UAE represents an environmentally friendly, efficient and rapid method for the extraction of flavonoid glycosides and triterpenoid saponins from licorice. The extract solution could be directly used for HPLC quantitative analysis for quality control of licorice. In addition, when the target compounds will be applied to medicine, ionic liquids can be removed through some simple chromatographic methods such as solid-phase extraction (SPE) or size exclusion chromatography (SEC).

### 3.3 Method validation

To evaluate the proposed IL-UAE method, linearity, stability, precision, repeatability and recovery were respectively investigated under the optimized conditions.

A series of reference solutions of LA, LQ, ILA, ILQ and GA at ten concentration levels were prepared to assess the linearity of the proposed method, and the working solutions were analyzed in duplicate to establish the calibration curves. The calibration curves were constructed by plotting the mean peak areas of the samples against the concentration of each compound. As shown in Table 1, all showed good linearity with correlation coefficients (*r*^2^) no less than 0.99, and LOQs (S/N = 10) and LODs (S/N = 3) for the five analytes are less than 0.321 μg mL^−1^ and 0.067 μg mL^−1^, respectively.

**Table 1:** Calibration curve, detection and quantification limits, precision, stability and repeatability for the five standard compounds

Analytea^a^	Calibration curve	*r* ^2^	LOD (μg mL^−1^)	LOQ (μg mL^−1^)	Intraday precision (*n* = 6)^b^	Interday precision (*n* = 6)^b^	Stability (*n* = 3)^b^	Repeatability (*n* = 6)^b^
LA	*y* = 956.4*x* + 40.718	0.9907	0.067	0.321	2.35	0.72	0.20	2.14
LQ	*y* = 1276.3*x* + 38.914	0.9971	0.051	0.242	7.38	0.07	0.91	1.36
ILA	*y* = 6733.5*x* + 34.495	0.9956	0.004	0.024	0.33	0.12	0.42	0.98
ILQ	*y* = 10626.0*x* + 40.582	0.9975	0.002	0.014	0.36	0.07	0.69	4.25
GA	*y* = 1637.5*x* + 99.245	0.9976	0.003	0.152	0.23	0.18	0.15	2.68

a LA, liquiritin apioside; LQ, liquiritin; ILA, isoliquiritin apioside; ILQ, isoliquiritin; GA, glycyrrhizic acid; LOD, limit of detection (S/N = 3); LOQ, limit of quantification (S/N = 10).

b Intraday precision, interday precision, stability and repeatability are expressed as the RSD (%) of peak area.

Intraday precision, interday precision, stability, repeatability and recovery of the optimized method were validated for each analyte. The analysis was repeated using the same sample six times in the same day, and additionally on six consecutive days to determine intra- and interday precision. Intra- and interday variations of the signals were less than 7.38% and 0.72% (represented by RSD values), respectively. The stability was evaluated by determining the sample solution at 0, 12, 24 and 36 h at 25 °C, and the variations of peak area expressed as RSD were 0.20%, 0.91%, 0.42%, 0.69% and 0.15% for LA, LQ, ILA, ILQ and GA, respectively, indicating that flavonoid glycosides and triterpenoid saponins were stable in IL aqueous solution for 36 h. To determine the repeatability of the IL-UAE method, six same samples were successively extracted. The extraction efficiency of five target compounds showed good repeatability with RSD values of 0.98–4.25%, suggesting that the proposed method had an acceptable level of repeatability (Table 1). Recovery tests were performed by adding known amount of each pure analyte into 0.05 g of licorice powder (*n* = 6). The developed analytical method showed acceptable recoveries with range from 98.69% to 101.68% (RSD < 3.54%, Table S2†). The method validation studies above indicated that the proposed extraction and analytical methods were credible.

### 3.4 Feedstock analysis to infer the extraction mechanism of IL-UAE

**Fig. 6:**
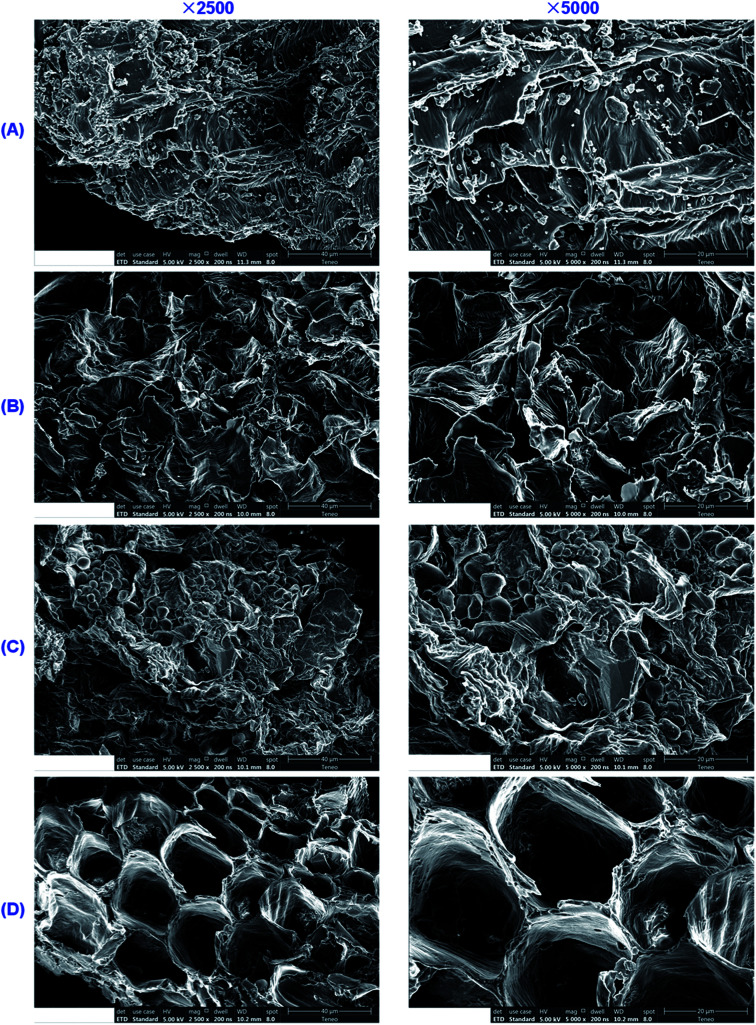
SEM graphics of licorice samples. (A) Raw materials; (B) treated by H_2_O-UAE; (C) treated by CH_3_OH-UAE; (D) treated by IL-UAE. The samples were observed under 2500× and 5000× magnification, respectively.

With the goal of inferring the reasons for the enhanced extraction of flavonoid glycosides and triterpenoid saponins by IL solutions, we applied scanning electron microscopy (SEM) to observe the untreated and treated licorice samples. The SEM images of the licorice samples before and after extraction using H_2_O, CH_3_OH and [C_4_MIM]Ac are shown in Fig. 6, and SEM images of licorice samples treated using the other ILs tested in the study are shown in Fig. S1.†Fig. 6A shows a micrograph of the raw materials, and the disruption of cells was rarely observed. However, in the samples treated with H_2_O-UAE, CH_3_OH-UAE and IL-UAE, cells and cell walls were affected by the broken effect of ultrasonic wave, thus leading to exposure of the target compounds to the extraction solution. Albeit broken cells were observed in all the three samples, the ratio of broken cells to intact cells remarkably increased in the presence of IL medium (Fig. 6B–D). The structures of samples treated with IL-UAE were broken completely, which contributed to the extraction of flavonoid glycosides and triterpenoid saponins into the IL aqueous solution. Hence, the extraction yields of LA, LQ, ILA, ILQ and GA were greatly improved in the presence of ILs since they permitted a better access to the target compounds embedded in the biopolymer matrix.

On the other hand, a lot of plant tissue powder was retained on the surface of the untreated samples (Fig. 6A), and small remnant amounts of the powder was retained in the samples that were subjected to CH_3_OH-UAE (Fig. 6C). As can be seen from Fig. 6B and D, however, the plant tissue powder on the surface was fully removed after H_2_O-UAE and IL-UAE. This might be due to stronger solvation of water and IL aqueous solution for target compounds in comparison with methanol.

## 4. Conclusions

In conclusion, [C_4_MIM]Ac was successfully used as the green solvent for IL-UAE of LA, LQ, ILA, ILQ and GA from licorice. The optimized IL-UAE conditions were as follows: extraction solvent 1.5 M [C_4_MIM]Ac aqueous solution, extraction time 20 min, solvent to solid ratio 10 : 1 mL g^−1^ and soaking time 8 h. Compared with conventional UAE, the proposed approach could result in better damage of licorice microstructures, and thus provided much higher extraction efficiency. Moreover, IL-UAE needed shorter extraction time and much smaller solvent to solid ratio than the pharmacopoeia method. To alleviate environmental pressure and to develop green sample preparation techniques, ILs as solvents in the UAE of flavonoid glycosides and triterpenoid saponins from licorice show a great promising prospect.

## Conflicts of interest

There are no conflicts to declare.

## Supplementary Material

RA-008-C8RA01056K-s001
