# Supplementary material for: Ionic liquids-ultrasound based efficient extraction of flavonoid glycosides and triterpenoid saponins from licorice
Source: RSC Adv. 2018 Apr 16;8(25):13989–96. doi: 10.1039/c8ra01056k (PMC9079881; doi:10.1039/c8ra01056k)
Supplement: RA-008-C8RA01056K-s001 [file RA-008-C8RA01056K-s001.pdf]

Electronic Supplementary Material (ESI) for RSC Advances.
This journal is © The Royal Society of Chemistry 2018

Ionic liquids-ultrasound based efficient extraction of flavonoid glycosides and triterpenoid saponins from licorice

Shuai Ji [1,2,#], Yujie Wang [1,#], Zhenyu Su [1], Dandan He [1], Yan Du [1], Mengzhe Guo [1,2], Dongzhi Yang [1,2], Daoquan Tang [1,2,*]

[1] *Department of Pharmaceutical Analysis, School of Pharmacy, Xuzhou Medical University, 209 Tongshan Road, Xuzhou 221004, China*

[2] *Jiangsu Key Laboratory of New Drug Research and Clinical Pharmacy, School of Pharmacy, Xuzhou Medical University, 209 Tongshan Road, Xuzhou 221004, China*

[#] The first two authors (S. Ji and Y. Wang) contributed equally to this paper.

[*] Corresponding authors. Tel.: +86 516 83263133. Fax: +86 516 83263133. Email address: tdq993@hotmail.com or tangdq@xzhmu.edu.cn.

Table S1. Names and structures of ionic liquids used in this study.

Names	Abbreviations	Cations	Anions
1-butyl-3-methylimidazolium acetate	$[C_4MIM]Ac$	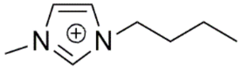	Ac^-
1-butyl-3-methylimidazolium chloride	$[C_4MIM]Cl$	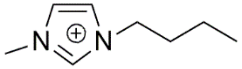	Cl^-
1-butyl-3-methylimidazolium tetrafluoroborate	$[C_4MIM]BF_4$	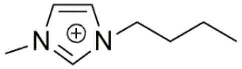	BF_4^-
1-butyl-3-methylimidazolium nitrate	$[C_4MIM]NO_3$	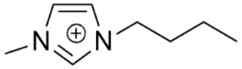	NO_3^-
1-butyl-3-methylimidazolium hydrogen sulfate	$[C_4MIM]HSO_4$	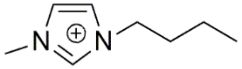	HSO_4^-
1-ethyl-3-methylimidazolium bromide	$[C_2MIM]Br$	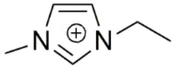	Br^-
1-butyl-3-methylimidazolium bromide	$[C_4MIM]Br$	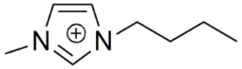	Br^-
1-hexyl-3-methylimidazolium bromide	$[C_6MIM]Br$	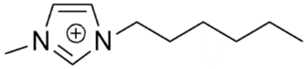	Br^-
1-octyl-3-methylimidazolium bromide	$[C_8MIM]Br$	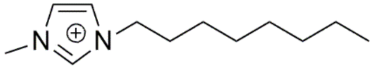	Br^-
1-decyl-3-methylimidazolium bromide	$[C_{10}MIM]Br$	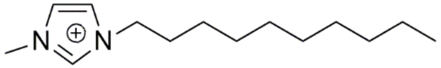	Br^-

Table S2. Recoveries of LA, LQ, ILA, ILQ and GA from licorice.

Analyte	Original (mg)	Added (mg)	Found (mg)	Recovery (%)	Mean (%)	RSD (%)
LA	3.09	3.04	6.00	95.78	101.51	3.54
	2.79	3.05	5.98	104.91		
	2.72	3.05	5.90	104.32		
	2.78	3.07	5.94	102.86		
	2.86	3.08	5.89	98.50		
	2.72	3.08	5.88	102.67		
LQ	4.91	4.06	8.94	99.09	101.19	2.95
	4.93	4.06	8.89	97.44		
	4.75	4.08	8.80	99.31		
	4.63	4.10	8.85	102.73		
	4.54	4.10	8.79	103.65		
	4.46	4.11	8.77	104.94		
ILA	0.67	0.59	1.25	97.15	101.68	2.67
	0.65	0.59	1.24	99.80		
	0.62	0.59	1.23	102.12		
	0.62	0.60	1.24	103.06		
	0.60	0.60	1.23	103.88		
	0.59	0.60	1.22	104.09		
ILQ	0.54	0.61	1.13	97.25	100.04	2.00
	0.54	0.61	1.14	98.46		
	0.53	0.61	1.13	99.22		
	0.51	0.61	1.14	102.17		
	0.50	0.61	1.13	101.53		
	0.50	0.62	1.12	101.57		
GA	12.21	9.09	20.95	96.10	98.69	3.12
	12.09	9.10	20.84	96.14		
	11.89	9.13	20.61	95.50		
	11.54	9.17	20.81	101.09		
	11.29	9.22	20.60	101.04		
	11.08	9.26	20.56	102.28		

Note: LA, liquiritin apioside; LQ, liquiritin; ILA, isoliquiritin apioside; ILQ, isoliquiritin; GA, glycyrrhizic acid.

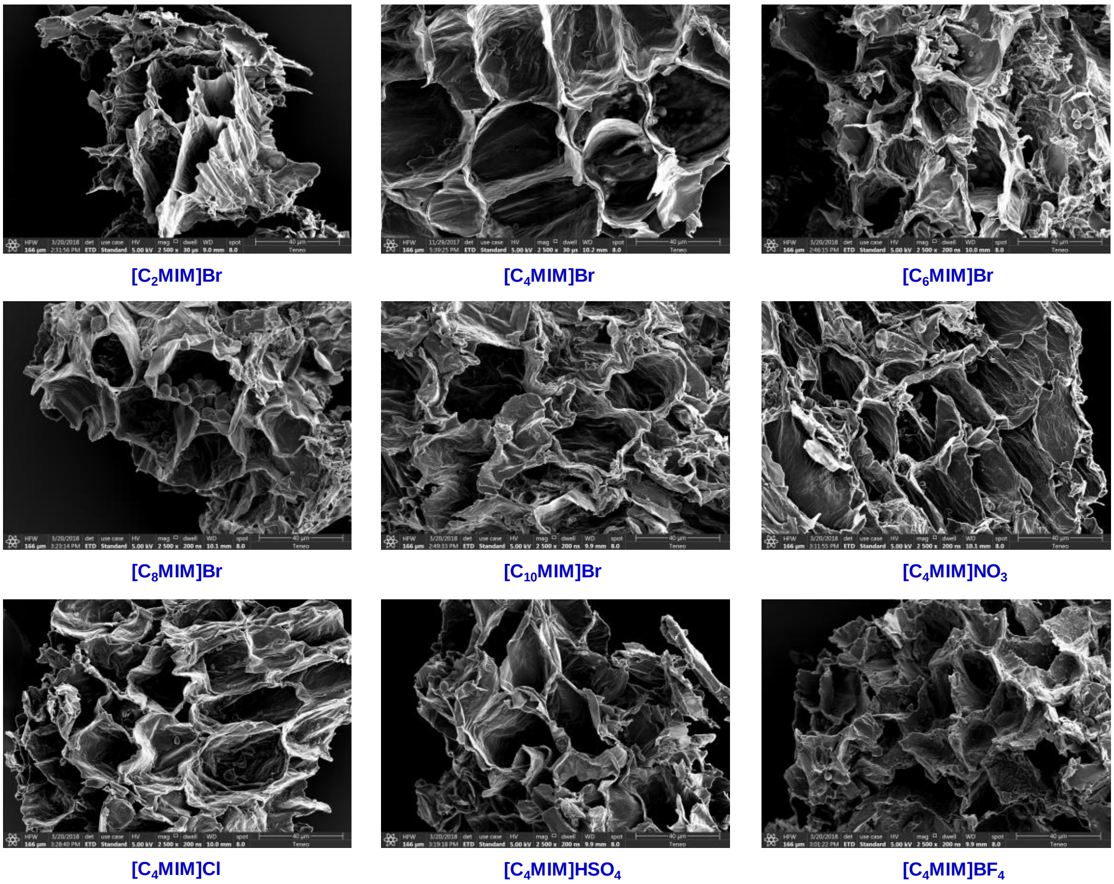

Fig. S1. SEM graphics of licorice samples treated using different solvents. The samples were observed under 2500 × magnification.
